# Advancing personalized spinal muscular atrophy care: matching the right biomarker to the right patient at the right time

**DOI:** 10.1007/s00415-025-13314-7

**Published:** 2025-09-02

**Authors:** Stefania Corti, Linda Ottoboni, Valeria Sansone

**Affiliations:** 1 Dino Ferrari Centre, Department of Pathophysiology and Transplantation (DEPT), Università Degli Studi Di Milano, Milan, Italy; 2 SC Neurology Unit, Department of Neurosciences and Mental Health, Fondazione IRCCS Ca’ Granda Ospedale Maggiore Policlinico, Milan, Italy; 3 SSD Neuromuscular and Rare Diseases, Department of Neurosciences and Mental Health, Fondazione IRCCS Ca’ Granda Ospedale Maggiore Policlinico, Milan, Italy; 4 NeMO Clinical Center, Milan, Italy; 5 Department of Neurorehabilitation, University of Milan, Milan, Italy

**Keywords:** Spinal muscular atrophy, Biomarker, Neurophysiology, Neurofilament, Prognosis, Monitoring

## Abstract

With the advent of survival motor neuron (SMN)-enhancing therapies, the natural course of spinal muscular atrophy (SMA) has been reshaped, unveiling new patient phenotypes. As therapeutic options expand, there is an increasing demand for robust biomarkers to enhance prognostic accuracy, anticipate treatment response, track disease progression, and support personalized clinical decision-making. This narrative review critically examines the literature and discusses the role and appropriate application of key biomarkers across different age groups, ranging from presymptomatic newborns to adults with chronic disease. Genetic testing remains the diagnostic gold standard, with *SMN2* copy number serving as the strongest prognostic indicator. However, substantial phenotypic variability exists among individuals with the same *SMN2* copy number. Neurophysiological measures, including compound muscle action potential (CMAP) and motor unit number estimation (MUNE), accurately inform about motor neuron integrity, often anticipating clinical changes and potentially predicting treatment responsiveness. Circulating neurofilaments (NF) are increasingly recognized as sensitive biomarkers of active neurodegeneration. While NF holds promise in infants and younger children, its relevance in adolescents and adults remains limited. Conversely, quantitative muscle imaging techniques, such as MRI and ultrasound, may be valuable tools in adolescent and adult patients, capturing long-term muscle structural changes. By reviewing the current evidence across age groups, we provide an overview of biomarker application in newborns, children and adolescents/adults for diagnostic, prognostic, predictive, and monitoring purposes to help advance individualized management across all SMA stages.

## Introduction

Spinal muscular atrophy (SMA) is a rare neuromuscular degenerative disorder characterized by the progressive loss of motor neurons and consequent muscle atrophy [1, 2]. It is an autosomal recessive genetic disorder primarily caused by homozygous deletions (or, less frequently, mutations) in the *SMN1* gene, which encodes for the survival motor neuron (SMN), an essential protein for the survival of motor neurons [3]. The disease presents with continuous and incremental symptom severity, and patients are typically classified into four main types (0–IV) according to age of onset, symptom severity and motor milestones achievement. Type 0 SMA is embryonically lethal, while type IV represents the mildest form, characterized by mild muscle weakness, while types I–III show a range of symptom severity [4–9].

The loss of functional SMN protein is partially compensated by the presence of *SMN2*, a paralogous gene of *SMN*. Compared to *SMN1*, *SMN2* carries a single nucleotide substitution (C > T) in exon 7, which causes aberrant splicing in the pre-mRNA. As a result, most *SMN2* transcripts skip exon 7, yielding a truncated SMN protein (SMNΔ7). Despite the altered *SMN2* splicing pattern, approximately 10% of *SMN2* transcripts include exon 7 and produce functional SMN protein [10, 11]. Overall, the amount of SMN functional protein expressed in patients with SMA varies depending on the individual's number of *SMN2* copies [11–14].

Recent years have witnessed groundbreaking advancements in the treatment of this disease. Three transformative, gene-targeting therapies have been approved, each aimed at increasing the availability of SMN protein: the splice-modifying drugs nusinersen and risdiplam, acting on *SMN2* pre-mRNA splicing to favor exon 7 inclusion, and onasemnogene abeparvovec (AVXS-101), a gene therapy delivering *SMN1* transgene through an adeno-associated virus (AAV9) vector [15–19]. Beyond SMN-targeting therapies, SMN-independent therapeutic strategies are also under investigation. These emerging approaches aim to modulate alternative pathways involved in muscle function, neuromuscular junction integrity, neuroprotection, cell survival, and epigenetic regulation. When used in combination with the approved SMN-enhancing treatments, these novel molecules may provide complementary benefits by addressing distinct pathophysiological mechanisms [20]. The advent of these SMN-enhancing therapies has substantially improved outcomes across all SMA severities, especially in presymptomatic infants and children [21, 22], younger patients [23], and patients with some residual motor function [24]. With the introduction of these treatments, the natural history of SMA has undergone a profound transformation, unveiling novel SMA phenotypes which differ across age of onset and show different residual symptom severity and motor milestone achievement [25]. Upon treatment, some patients initially categorized as type I or type II have achieved unexpected milestones for their SMA type, thereby exceeding the limits of the classification system [26]. As a result, the traditional classification of SMA into types 0–IV has become increasingly outdated and less reflective of patient outcomes, based on functional status [26–29]. To overcome the barriers associated with this classification, patients are now referred to as non-sitters, sitters, and walkers, thus allowing the inclusion of new phenotypes emerged with the introduction of the new treatments.

Because of the effective delay in the progression of SMA, many patients, including adults with severe disabilities and parents or caregivers of pediatric patients, report meaningful improvements following treatment. However, profound inter-individual variability in treatment response has been observed in patients despite similar baseline characteristics [30, 31]. Growing evidence from clinical trials and real-world evidence [25, 32–48] provides refined clinical information and relatively adequate data on the advantages and limitations of each treatment available. However, the degree of variability between patients underscores the need for additional information to guide clinical practice. In general, there is consensus that the number of *SMN2* copies, the initial level of disability when treatment is started, and the age at which this occurs are the most significant determinants of the outcome. Yet, this information does not fill the gap that still exists when considering treatment choices, prognosis, and patient trajectories over time. In some cases, therapies stabilize the patient's condition, preventing further decline in function, while in other cases, they may lead to significant improvements, by either partially restoring muscle functions or enhancing overall motor performance. Systemically administered therapies such as risdiplam and AVXS-101 may have effects beyond the central nervous system. Indeed, it is not unusual to see that impact on neuromotor, respiratory, or swallowing functions, which may follow different timelines. Conversely, nusinersen is expected to have primarily CNS-mediated effects, due to its intrathecal administration and pharmacokinetic properties. Nevertheless, preliminary evidence suggests that systemic outcomes may also occur with this molecule [49–51]. Importantly, in a relentlessly progressive disease, such as SMA, and considering that current treatments may not completely block disease progression, maintaining stability is increasingly recognized as a meaningful therapeutic success. This leads to a discussion of patients’ expectations, which is crucial in guiding care and management.

The development of multimodal assessments, ideally tailored to the unique characteristics of different patient populations, is becoming increasingly necessary to fully capture the diverse therapeutic responses to these treatments. While standardized tools remain valuable, they often lack the sensitivity to detect subtle but meaningful changes in disease progression or improvements in less commonly measured functional domains [52]. This is often an additional limitation in the clinic to guide treatment choices and manage expectations.

In this regard, the integration of multiple biomarkers is anticipated to play a central role, serving different purposes throughout the course of the disease. Diagnostic biomarkers are essential for early detection and confirmation of the disease, which is particularly crucial in presymptomatic cases identified through newborn screening (NBS). Prognostic biomarkers predict symptom severity and disease progression, while predictive biomarkers help identify patients most likely to benefit from specific interventions. Pharmacodynamic biomarkers enable the assessment of treatment responses and may support clinicians in ongoing management during follow-up monitoring [53, 54].

This narrative review examines the evolving role of selected biomarkers (genetic biomarkers, neurophysiological measures, neurofilaments, and imaging techniques) in guiding clinical decision-making and supporting patients throughout the SMA journey, from diagnosis to treatment and long-term monitoring. These biomarkers have been selected because they are routinely used in clinical practice and/or are supported by a substantial body of evidence in the literature. Other biomarkers have not been included either because they are not commonly used in clinical practice or because data about their long-term clinical significance are limited.

To illustrate the practical application of these biomarkers in clinical practice, we present a series of hypothetical clinical case studies. By providing pragmatic examples, we show how biomarkers can empower clinicians and patients, address unmet needs, and shape the future of SMA care.

## The role of SMA biomarkers in presymptomatic newborns

### Genetic biomarkers

In presymptomatic newborns, diagnosis depends exclusively on genetic testing to identify deletions or mutations in the *SMN1* gene through NBS [55–57]. Given the demonstrated effectiveness of current therapies in altering the disease course, particularly when initiated early, population-wide screening programs and extended access to genetic testing to search for *SMN1* gene alterations have become increasingly available. Indeed, treatment initiation during the presymptomatic phase is associated with improved outcomes, enhanced long-term prognosis, and the potential to substantially alter the course of the disease [22, 27, 29, 58].

Once a presymptomatic newborn is diagnosed with SMA through NBS, knowing the *SMN2* copy number becomes essential for prognosis. Although limited, the production of functional SMN protein from the *SMN2* gene plays a significant role in modulating disease severity and progression [59, 60]. In general, a higher number of *SMN2* copies is associated with a milder clinical phenotype. Patients with the most severe form, type 0 SMA, typically have a single *SMN2* copy, while those with type III and IV SMA have three or four copies [13, 55, 61–65]. As such, the *SMN2* copy number currently stands as the strongest prognostic biomarker in SMA, especially in presymptomatic patients [27].

However, emerging evidence indicates that identical *SMN2* copy numbers can be seen across patients with varying degrees of symptom severity, SMA types and progression rates [64, 66–69], suggesting that the relationship between *SMN2* copy number and phenotype is more complex than initially perceived [70–72]. The study by Ricci et al. [68] investigated the clinical phenotypes of pediatric and adult patients who possessed four copies of the *SMN2* gene. Among 169 patients, most (66%) were classified as type IIIb SMA, with smaller proportions having type IIIa (24%) and 5% II or IV. Despite the presence of four *SMN2* copies, patients displayed a wide range of SMA types and clinical severity. Functional decline was common, with loss of ambulation occurring in 35% of type III and 25% of type IV patients. However, the cohort also included six presymptomatic individuals and two asymptomatic adults. By highlighting the considerable phenotypic variability in disease progression within this genetic subgroup, these findings indicate that relying solely on *SMN2* copy number to predict disease severity may be insufficient [68]. Potential modifiers of disease severity have been identified and include, among others, *SMN2* gene variants, such as the c.859G > C variant, associated with the mildest phenotypes [59]; Plastin 3 (PLS3), which acts as a protective modifier particularly in females [73, 74]; and neurocalcin delta (NCALD), whose reduction protects against SMA by restoring impaired endocytosis [75]. These modifiers may contribute to the phenotypic variability observed among patients with identical *SMN2* copy numbers.

### Neurophysiological measurements in presymptomatic newborns

The use of supplementary biomarkers beyond genetic testing for *SMN1* and *SMN2* copies becomes increasingly important, especially in presymptomatic newborns diagnosed with SMA through NBS. Although long-term data (with follow-up of up to 5 years in treated presymptomatic children [36]) suggest that motor function is maintained, additional measurements over time are necessary to confirm the extent to which early treatment prevents phenoconversion, and assess the “health” of the motor neuron pool. Among neurophysiological assessments, compound muscle action potential (CMAP) amplitude effectively informs about motor neuron integrity and predicts disease progression [76, 77]. Its values vary according to SMA type and *SMN2* copy number [76] and correlate with motor function [78, 79]. CMAP amplitude can enhance initial clinical assessment [80], as reduced values often precede the onset of overt motor symptoms [55, 81].

### Neurofilaments in presymptomatic newborns

In addition to neurophysiological measures, there is consensus that neurofilaments (NFs) can be considered as biomarkers of active neurodegeneration; upon cellular damage, NFs are released from neurons and can be found in both cerebrospinal fluid (CSF) and blood [82, 83]. Indeed, elevated levels of phosphorylated NF-heavy chain (pNF-H) and NF-light chain (NF-L) have been found in several neurologic disorders [83–86], including SMA [87]. Nowadays, there is significant interest within the scientific community in advancing the understanding and utilization of NFs as a prognostic and predictive biomarker in patients with SMA [54]. Although not routinely performed in clinical practice, measurement of NF levels may complement the prognostic information obtained from genetic and neurophysiological biomarkers. Notably, serum pNF-H and NF-L concentrations have been shown to correlate with *SMN2* copy number, earlier age of symptom onset, and CMAP amplitude values [88]. Beyond their sensitivity in detecting active neurodegeneration and identifying subclinical pathological changes, measuring NF levels offers added value through their emerging role in predicting response to treatment. In general, both blood and CSF NF levels are higher in SMA newborns compared to healthy controls [87, 89, 90], and both decline following nusinersen treatment [87, 89]. A strong correlation has been reported between serum and CSF NF-L levels in pediatric patients [90]. Given their less invasive nature, blood-based NF measurements are generally preferred in clinical practice. However, when nusinersen is the selected treatment, its intrathecal administration can be leveraged to assess NF levels in the CSF.

The NURTURE study [89] was pivotal in revealing that plasma pNF-H levels at baseline and day 64 after nusinersen initiation effectively predicted both Hammersmith Infant Neurological Examination - Section 2 (HINE-2) total motor milestone achievement and early independent walking. Compared with CMAP amplitude and age, pNF-H was the strongest predictor of motor function [89]. While this study suggests that NFs may be considered useful biomarkers for predicting the response to nusinersen, this does not seem to be applicable to predict outcomes using other treatments, such as AXVS-101. After dosing in patients started on gene therapy, NFs show a rapid, transitory increase [88, 91]. Intriguingly, the rise in NF-L was minor or null in infants who were pre-treated with nusinersen before AXVS-101 administration. The initial increase in NF levels could be due to an inflammatory response triggered by the infection of neuronal cells by AAV9 particles [91]. No data are currently available on the effects of risdiplam on NF levels [88].

The key studies assessing the role of biomarkers in newborns are summarized in Table 1 [55, 57, 80, 81, 88, 89].

**Table 1:** Main studies assessing the role of biomarkers in newborns

Study (first author, year)	Study design	Number of patients	Age	Biomarker(s)	Role	Main findings
Vill et al., 2019 [55]	Prospective newborn screening study	165,525 screened; 22 SMA cases identified	Newborns (median treatment initiation: 24 days)	*SMN1* exon 7 deletion, *SMN2* copy number, CMAP amplitude	Diagnostic, Prognostic, Predictive	Early diagnosis through NBS led to presymptomatic treatment. *SMN2* copy number and CMAP values correlated with disease severity; low CMAP values preceded motor function decline
Kariyawasam et al., 2020 [80]	Prospective newborn screening study	103,903 screened; 9 SMA cases identified	Newborns	*SMN1* exon 7 deletion, *SMN2* copy number	Diagnostic, Prognostic	Early diagnosis through NBS enabled early treatment initiation. Lower *SMN2* copy numbers correlated with earlier symptom onset and more severe phenotypes
Berzal-Serrano et al., 2025 [57]	Newborn screening study	31,560 screened; 4 SMA cases identified	Newborns	*SMN1* exon 7 deletion, SMN2 copy number	Diagnostic, Prognostic	*SMN1* deletion confirmed SMA diagnosis; *SMN2* copy number guided early treatment decisions
De Vivo et al., 2019 [89]	Phase II clinical trial	25	≤ 6 weeks old at first nusinersen dose	NF (pNF-H), SMN2 copy number	Prognostic, Predictive	Early nusinersen treatment improved motor function; NF levels declined with treatment
Weng et al., 2021 [81]	Newborn screening study	21	Newborns	CMAP amplitude, *SMN2* copy number	Predictive, Prognostic	CMAP drop predicted symptom onset; higher pretreatment CMAP correlated with better response to nusinersen
Alves et al., 2021 [88]	Longitudinal cohort study	68 untreated, 22 treated	0–3 years (range)	NF (pNF-H, NF-L)	Prognostic, Predictive	Higher NF levels were associated with disease severity; nusinersen reduced NF levels, but onasemnogene abeparvovec increased them

*CMAP* Compound muscle action potential, *MRI* Magnetic resonance imaging, *MUNE* Motor unit number estimation, *MUNIX* Motor unit number index, *MUSIX* Motor unit size index, *NF-L* Neurofilament light chain, *pNF-H* Phosphorylated neurofilament heavy chain, *SMA* Spinal muscular atrophy, *SMN* Survival motor neuron

### Case study 1

A. was the first child born to parents who were unaware of being carriers of a *SMN1* deletion, in a country where NBS is available. After 72 h from dried blood spot (DBS) screening, confirmation of *SMN1* deletion was available, and three *SMN2* copies were detected. On day 3, A. was seen by a multidisciplinary team involving a neurologist, a child neurologist, a neonatologist, and a pediatrician, and the diagnosis was discussed with the parents. Data from clinical trials and real-world evidence were presented to contextualize the prognosis and treatment options. CMAP was also discussed as important to define motor neuron integrity. After having obtained informed consent from both parents, the ulnar nerve CMAP was tested and provided values within the normal range (amplitude > 5.9 mV, distal latency < 3.8 ms for age). A blood sample was obtained for anti-AAV9 antibodies and was also stored for future testing, including NFs levels. Treatment options were discussed with the parents. Follow-up was planned for 1 week later or as needed, based on clinical signs that may arise, and treatment was started.

In this case, the clinical status, the number of *SMN2* copies, and the CMAP values were the key determinants used to discuss the prognosis, the treatment options, and expected outcomes, based on available evidence.

### Case study 2

B. was born to parents who were known SMA carriers and was the second child in the family. The family resided in a country where NBS was not available. The older sibling had received the diagnosis of SMA at 3 years of age, manifesting progressive lower limb weakness and reduced walking abilities. He was found to have four *SMN2* copies. Blood was collected to assess the level of anti-AAV9 antibodies and stored for additional testing. CMAP testing of the ulnar nerve was performed, and values were within the normal range. Parents decided to proceed with treatment. Follow-up was planned based on the selected treatment and would be adapted based on any emerging clinical symptoms.

In this case, the clinical status, the number of *SMN2* copies, the CMAP values, and the sibling’s phenotype were the main factors used to discuss the prognosis, the most appropriate treatment, and the expected outcomes, based on available evidence.

### Case study 3

C. was born in a country where NBS is available. Parents were unaware of being carriers of an *SMN1* deletion. On day 3, genetic test results confirmed SMA in the newborn; C. presented only one copy of *SMN*2. The high risk of rapid disease progression and potentially life-threatening symptoms was clearly communicated to the family. C. was admitted for close clinical monitoring and initiation of a treatment plan. Anti-AAV9 antibody levels were tested, and a blood sample was stored for future testing, including the quantification of exploratory biomarkers. CMAP amplitude of the ulnar nerve was measured and found to be below the normal threshold for age. Treatment was discussed and planned based on clinical status, prioritizing the stabilization of the disease and implementation of standard of care (SoC) in response to any emerging symptom of neurodegeneration.

In this case, the clinical status and the number of *SMN2* copies were critical to discuss the need for careful monitoring, prioritizing clinical management and pharmacological treatment as an “add-on” treatment to improve prognosis and expected outcomes, based on available evidence. Neurophysiology data were acquired as a potential tool to monitor disease progression and to support long-term clinical decision-making.

## The role of SMA biomarkers in children

Studies focusing on children with SMA cover a broad age range, spanning from a few months to 12 years of age; unsurprisingly, this patient's setting exhibits a wide spectrum of disease severity. This heterogeneous population presents unique challenges, as the trajectory of disease progression differs considerably depending on multiple factors, including the time of treatment initiation and overall disease severity. Most children with type I, II, and III SMA who have already developed symptoms are currently receiving treatment, and their response varies widely.

### Neurophysiological measurements in children

Neurophysiological tests can help reveal subclinical changes in motor units, which may not necessarily correspond to subjective and objective measures of motor function. Among these, CMAP and motor unit number estimation (MUNE) have been widely studied; other measurements, such as motor unit number index (MUNIX) and motor unit size index (MUSIX), may provide additional information [92, 93].

CMAP measurement may serve to assess disease severity and predict disease progression and treatment response. In children, CMAP amplitudes vary according to disease severity and correlate with motor function [77, 94], though variability exists across SMA types [95–97]. Nusinersen increases CMAP amplitudes over time, consistently with improved motor function [94, 98]. Higher baseline CMAP values have been associated with better treatment outcomes for both nusinersen and AVXS-101 [94, 99–101]. Greater improvements in CMAP amplitudes may be observed in younger children and those receiving early treatment, possibly because they retain more viable motor units than older patients or children receiving treatment at an older age [98, 99, 102].

MUNE is considered more sensitive than CMAP for detecting subtle changes in motor unit loss or reinnervation, as it identifies changes at the level of individual motor units [94, 103]. Like CMAP, MUNE values also generally correlate with *SMN2* copy number and vary according to SMA types [76, 94, 97, 103, 104]. MUNE also correlates with motor function, although the strength of this association varies. This variability is likely due to MUNE's ability to detect"silent"denervation, where the significant motor unit loss occurs without noticeable declines in motor function, which is possibly maintained by compensatory mechanisms, such as nerve sprouting and enlargement of surviving motor units [97]. Interestingly, Kang et al. observed a longitudinal increase in MUNE values in treatment-naïve patients, especially those aged 5–10 years old, suggesting spontaneous motor unit development [97]. This is in contrast with the study by Swoboda et al., who also considered untreated patients but reported a progressive decline in MUNE values over time. Several factors may explain this discrepancy, including differences in both the age of patients at the start of longitudinal monitoring and the proportion of patients with SMA type I, II, and III [76, 97]. In children treated with nusinersen, MUNE is often one of the earliest neurophysiological measures to show improvement following treatment initiation. The magnitude of this initial increase is associated with a greater likelihood of achieving clinically meaningful motor function gains [94]. MUNE is a valuable tool for tracking disease progression in SMA, especially in early or less severe stages where motor function is preserved and CMAP may fail to detect meaningful changes. However, MUNE values should be interpreted with caution as multiple variables, including patients'age, disease duration, extent of denervation, and types and locations of nerves tested, may affect MUNE changes over time.

Other neurophysiological parameters derived from CMAP measurement, including N50, A50, and largest single motor unit potential (LSMUP), should be evaluated in children alongside CMAP and MUNE, as they offer insights into motor unit remodeling and reinnervation. In the pivotal study by Kariyawasam et al., N50 values increased over time in children treated with nusinersen, suggesting that denervation is reduced within larger motor unit pools, and the extent of N50 improvement from baseline was associated with a higher probability of motor function improvement. In contrast, A50 and LSMUP, surrogates of collateral reinnervation, did not change [94].

### Neurofilaments in children

A substantial body of evidence supports the multiple roles of NFs in infants and young children with SMA, including their use as diagnostic, prognostic, predictive, monitoring, treatment response, and potential risk biomarkers [87, 89, 90, 105, 106]. NF levels are elevated in pediatric patients compared to age-matched healthy controls [90, 106, 107]; they also progressively decline with age and vary according to SMA severity, disease duration, and are usually higher in the acute than chronic phase [88, 90, 107, 108]. As they influence the baseline levels of NF, age and disease severity are also the main factors that determine the extent of NF changes upon nusinersen treatment. Overall, nusinersen reduces NF-L levels in both the CNS and serum, with the most significant decline occurring in children with the most severe SMA phenotypes [88, 90, 106, 107, 109]. Given this growing body of literature, the European Medicines Agency (EMA) has endorsed the potential use of NF-L as a biomarker for pediatric neurological diseases, including SMA, emphasizing its importance for clinical management and research [110]. As elevated NF-L levels reflect disease activity and can be considerably decreased by nusinersen treatment [90, 107], they can serve as valuable indicators for guiding therapeutic decisions, particularly regarding the timing of intervention. Although further research is needed to determine the most suitable biomarker – whether NF-L or pNF-H – the optimal biological fluid (CSF or plasma), and the most appropriate patient subset (children with SMA type I or all children with ≤ 2 *SMN2* copies), NF remains a promising biomarker that could indicate therapeutic success even in the absence of obvious motor unit score improvement.

### Imaging techniques in children

Imaging techniques, including muscle ultrasound and magnetic resonance imaging (MRI), can inform about the status of muscle composition and/or architecture in SMA patients. In children, muscle pathology is dynamic and rapidly changing, making it harder to capture meaningful changes with static imaging. Although the role of muscle imaging in tracking SMA progression is still unclear, ultrasound and MRI have been shown to discriminate against children with SMA based on disease severity, thus constituting a potential biomarker for disease monitoring [111–116]. Quantitative MRI applied to children with SMA types II and III receiving nusinersen showed an increase in the fat fraction (FF) in the majority of tight muscles. Despite the progressive fat infiltration, children showed clinical improvement. In contrast to FF, improvements in other exploratory parameters obtained from diffusion tensor imaging (DTI) were observed, highlighting a potential role for these parameters as biomarkers to follow treatment outcomes. Although preliminary, these data may suggest that nusinersen may positively affect muscle microstructure, potentially decreasing muscle atrophy and restoring abnormal but viable muscle rather than preventing fat infiltration [117]. Despite these encouraging findings, the rapid neurodegeneration taking place in children with SMA makes neurophysiological parameters and NF measurement more informative biomarkers than muscle imaging techniques to assess disease activity. Moreover, MRI is both time and cost intensive, further justifying the use of neurophysiological parameters and NF level measurement in this age group in routine clinical practice.

### Clinical significance of biomarkers in pediatric patients

As discussed above, biomarker values tend to fluctuate with age, possibly indicating different scenarios (e.g., acute neurodegeneration, motor neuron preservation, compensatory reinnervation, etc.) in patients within the same age category. Therefore, the clinical relevance of each SMA biomarker may differ between early and late childhood; the use of biomarkers should be tailored according to the child's age and result interpretation requires great caution due to underlying heterogeneity in disease stage and prior treatment history. In the pediatric setting, the primary treatment goal should be symptom improvement, particularly in young children diagnosed and treated early, but again, this is highly dependent on the baseline clinical characteristics of the child, especially if respiratory and swallowing functions are affected.

Discussion on expectations is a crucial step in the care process and should be fully integrated into the treatment pathway. It deserves adequate recognition and sufficient time, as communication and active listening are fundamental components of the care and cure of each individual. In these discussions, it is important to explain that different domains may improve at different times (e.g., motor > respiratory muscles > swallowing function, in different orders) and may require different timelines. Data from clinical trials and real-world studies suggest that 6–12 months more are required to see more clinically significant changes [89, 94, 98]. These changes need to be weighed against what would have been the natural course without treatment, from previous literature studies. Potential achievements or progression should always be discussed based on expectations. Patients’ and families’ preferences should always be considered [118–120].

Recent consensus guidelines [7, 27] indicate that add-on or switch therapy should generally be considered only after a prolonged period of monotherapy, also considering country-specific regulatory approvals and reimbursement policies. Special consideration should be given to potential worsening and greater need for rehabilitation programs after spine surgery, which typically occurs at this stage [121–123].

The main studies assessing the role of biomarkers in children with SMA are reviewed in Table 2 [90, 94, 99, 101, 106, 107, 117].

**Table 2:** Main studies assessing the role of biomarkers in children

Study (first author, year)	Study design	Number of patients	Age	Biomarker(s)	Role	Main findings
Kariyawasam et al., 2021 [94]	Prospective cohort study	20	99 months (median) (range: 4–193)	CMAP, MUNE, N50	Prognostic Predictive	Baseline CMAP and MUNE correlated with *SMN2* copies and motor function. Nusinersen increased CMAP, MUNE, and N50 values. CMAP increases ≥ 4.5 mV and MUNE increases ≥ 15 units correlated with a higher likelihood of motor improvements in patients with type II and III SMA
Barrois et al., 2023 [101]	Prospective cohort study	25	227 days (median, Cohort 1) (range: 114–334) 247 days (median, Cohort 2) (range: 26–496)	CMAP amplitude	Predictive	CMAP combined with motor scores predicted motor improvement during treatment with AVXS-101
Richard et al., 2024 [99]	Longitudinal study	10	From 11 months to 10 years	CMAP amplitude	Predictive	Increased CMAP values correlated with motor improvements in SMA type II patients treated with nusinersen. Patients aged < 5 years showed more pronounced CMAP and motor improvements than older children
Olsson et al., 2019 [106]	Prospective cohort study	12 patients, 11 controls	14.4 months ± 24.0 (mean ± SD) (range: 1.2–92.4)	NF-L	Predictive, Monitoring	CSF NF-L levels decreased and correlated with motor improvement during nusinersen treatment in patients with SMA type I and two *SMN2* copies
Nitz et al., 2021 [90]	Observational study	18 patients, 97 controls	From 18 days to 17.2 years	NF-L	Predictive, monitoring	Serum NF-L levels decreased and correlated with motor function only in patients with up to two *SMN2* copies during nusinersen treatment
Seo et al., 2023 [107]	Biomarker study	8	From 1.1 to 270 months	NF-L	Predictive, monitoring	CSF and plasma NF-L levels decreased during nusinersen loading phase, followed by a plateau in patients with SMA type I and II
Otto et al., 2024 [117]	Longitudinal MRI study	8	9 years (mean) (range: 7.7–13.8)	Quantitative MRI	Monitoring	MRI detects ongoing fat infiltration and improvement in muscle microstructure during nusinersen treatment

*CMAP* Compound muscle action potential, *MRI* Magnetic resonance imaging, *MUNE* Motor unit number estimation, *NF-L* Neurofilament light chain, *SMA* Spinal muscular atrophy, *SD* Standard deviation, *SMN* Survival motor neuron

### Case study 4

D. was a 2 year-old child diagnosed with SMA type II at 8 months of age due to progressive motor weakness and delayed motor milestones. The child had three copies of *SMN2*. At diagnosis, neurophysiological studies revealed significant motor neuron loss: CMAP amplitude of the ulnar nerve was reduced (2.1 mV, below normal range for age), and MUNE showed only 15 motor units (normal > 40 for age). Blood was drawn for NF-L measurement, which showed elevated levels (85 pg/ml, above normal age range of 2.73–25.00 pg/mL). Treatment with a disease-modifying treatment was initiated. During follow-up at 6, 12, and 18 months, serial neurophysiological assessments provided evidence of motor neuron pool recovery through collateral sprouting mechanisms. While MUNE remained relatively stable, increasing modestly to 18 motor units, CMAP amplitude improved significantly to 6.8 mV. The key evidence for collateral sprouting came from motor unit remodeling parameters: A50 increased from 0.8 to 2.1 mV, and LSMUP increased from 2.5 to 4.8 mV, indicating that surviving motor neurons had significantly enlarged their innervation territories by sprouting to reinnervate orphaned muscle fibers. Additionally, single motor unit potential (SMUP) analysis showed increased amplitude and duration, confirming enlarged motor unit size. These neurophysiological changes preceded clinical motor improvements by several months, with the child eventually showing gains in motor function scores. At 18 months, NF-L levels had decreased to 28 pg/ml. This case demonstrates how detailed neurophysiological analysis can reveal compensatory reinnervation as the primary mechanism of functional recovery, where surviving motor neurons expand their innervation through collateral sprouting to partially compensate for motor neuron loss, providing valuable insights into treatment response mechanisms in pediatric SMA.

## The role of SMA biomarkers in adolescents and adults

Most patients with SMA who reach adolescence and enter adulthood have less severe SMA types [124] and are characterized by a chronic phase of gradual motor neuron loss and progressive muscle weakness. In contrast to younger children, where early intervention can substantially modify disease trajectory, treatment-related improvements in older SMA patients may take longer to manifest, likely due to the slower progression of the disease and the extent of pre-existing neuromuscular impairment. Therefore, the main goal is stabilization or reduction in the rate of progression in most functions. Evaluation of biomarker dynamics is pivotal to help stratify patients and depict treatment responses.

However, it is important to note that patients who reach adulthood may frequently present pronounced musculoskeletal deformities and neuromuscular impairment, which significantly limit the applicability of commonly used motor scales, such as the Hammersmith Functional Motor Scale – Expanded (HFMSE) and Revised Upper Limb Module (RULM). Many adult patients show either a floor or, more rarely, a ceiling effect on these scales. This highlights the importance of identifying reliable biomarkers capable of capturing subtle disease progression and treatment response that existing functional scales may not detect in this specific population.

### Neurophysiological measurements in adult patients

Like in other settings, neurophysiology techniques can be used to monitor adolescent and adult patients and may deepen our understanding of motor unit remodeling. Overall, reduced CMAP values appear to be more visible in the most damaged muscles. Using MScanFit, CMAP values from the abductor pollicis brevis (APB) muscle were similar between adult patients with type III SMA and healthy controls [125, 126]. However, these studies also showed that there was a reduction in D50, indicating substantial axonal loss, which was partially compensated by increased single motor unit amplitudes, suggesting that collateral sprouting occurs in adults with SMA [125]. Differently, in a population of pediatric and adolescent patients with SMA type II and III (either untreated or receiving nusinersen or risdiplam), CMAP and MUNE values from APB, abductor digiti minimi (ADM), and tibialis anterior muscles varied according to SMA type. In general, measures of collateral reinnervations (median amplitude and LSMUP) were lower in walkers compared to sitters [126]. These findings were similar to a previous study of adolescent and adult patients, in which CMAP amplitude, MUNE, and D50 values of median nerves significantly differed across SMA types, and more severe patients showed lower CMAP amplitudes and more pronounced collateral reinnervation [103]. Notably, adult patients exhibit a distinct pattern of motor unit degeneration, characterized by greater motor neuron loss in the ADM compared to the APB. This"reversed split-hand"phenomenon is specific to SMA [127]; as the APB muscle is relatively well preserved compared to the ADM, measurement of the median nerve CMAP amplitude may be a more suitable neurophysiological biomarker in this population. Neurophysiological parameters were shown to improve following nusinersen treatment, although with some discrepancies [114, 115]. In particular, ulnar CMAP and SMUP values, but not MUNE, improved in both ambulatory and non-ambulatory patients up to 14 months of treatment, implying that the changes in CMAP amplitudes may depend on collateral sprouting rather than an increase in the number of motor units [128, 129]. Recent studies have also investigated neurophysiological changes in adults treated with risdiplam. A significant increases in median nerve CMAP amplitude has been observed in 18 adult patients treated with risdiplam for 10 months, with improvements correlating well with clinical outcome scores [130].

MUNIX and MUSIX are promising biomarkers for characterizing the pattern of motor neuron loss in adults, being potentially more sensitive than traditional CMAP measurements in detecting motor neuron degeneration [128]. MUNIX values are strongly associated with disease severity [129, 131]; both MUNIX and MUSIX correlate with muscle strength and clinical disability scores [132]. Interestingly, the effects of nusinersen were evaluated in CMAP, MUNIX, and MUSIX from the orbicularis oculi muscle; no significant changes in any of the parameters were detected after 10 months, possibly because of the short duration of the observation period [133].

Overall, these findings highlight the challenges in monitoring disease progression and treatment response in patients with chronic SMA. Given the broad variability in terms of age, disease severity and duration, and retained motor function in SMA patients, alongside the different techniques used and the distinct muscles assessed in published studies, the results cannot be easily generalized. Nevertheless, neurophysiological measurements are essential to monitor changes in motor unit pools and evaluate the interplay between axonal loss and compensatory collateral sprouting at the individual patient level.

### Imaging techniques in adult patients

Disease progression may also be assessed via muscle MRI and ultrasound techniques in adolescent and adult patients. These imaging modalities appear to be more suitable for monitoring long-term, progressive muscle changes, effectively capturing atrophy, fatty infiltration, and fibrosis, which are more pronounced in advanced disease stages. MRI measurements from both upper and lower limb muscles significantly differed across SMA types and according to the walking ability of patients, with some variability depending on the proximal/distal level and type of muscles assessed [115]. In adult patients, FF in the tight muscles of patients with SMA types II and III significantly increased over time despite the lack of evident worsening in muscle strength and motor function scores. Exploratory DTI parameters such as fractional anisotropy (FA) and mean diffusivity (MD) were decreased and increased, respectively, compared with healthy controls and correlated with muscle strength and motor function [134, 135]. FF and DTI measures were also decreased in the upper muscles in SMA patients vs. healthy controls [136]. Other studies confirmed that MRI could assist in monitoring the slow disease progression in adults, emphasizing large variability in muscle involvement from proximal to distal regions across and within SMA types [113, 137]. Treatment with nusinersen did not affect FF values, despite parallel stabilization or improvement in motor function scores [138, 139]. However, while FF remained unchanged, nusinersen reduced baseline FA values in two siblings with SMA type IIIb after 10 months, with either stabilization or further decline at 24 months, implying that muscle atrophy may occur independently of FF [138]. Like MRI, muscle ultrasound can help assess the severity of muscle atrophy and structural changes in adult SMA, indicating its potential role as a disease severity marker [140]. More and larger studies are needed to further elucidate the role of MRI and ultrasound as reliable SMA biomarkers for adolescents and adults in clinical practice.

### Neurofilaments in adult patients

Differently from newborns and children, NF levels in adolescents and adults are generally comparable to those of healthy controls [141–146]. When measured before and after nusinersen treatment, the levels of plasma and/or CSF NFs (either NF-L or PNF-H) did not show meaningful variations despite improvements in clinical outcomes [141–143, 145–147]. Even if NF-L levels were recently found to be decreased during nusinersen treatment, this reduction did not consistently correspond to clinical improvements, and its clinical significance remains uncertain [148]. Given the current evidence, NFs are unlikely to represent a relevant biomarker in this patient setting.

Given the variability in age, disease duration, and individual disease burden, personalized interpretation of these biomarkers is critical for optimizing patient care.

Table 3 reports the main studies evaluating the role of biomarkers in adolescents and/or adults with SMA [103, 125, 126, 128, 129, 132, 134, 138].

**Table 3:** Main studies assessing the role of biomarkers in patient cohorts predominantly consisting of adolescents and/or adults

Study (first author, year)	Study design	Number of patients	Age	Biomarker(s)	Role	Main findings
Sleutjes et al., 2020 [103]	Cross-sectional study	24 patients types 2–4	39 years (median) (range: 12–75)	CMAP, MUNE, D50	Monitoring	CMAP, MUNE, and D50 values correlated with motor function scores and SMA severity
Elsheikh et al., 2021 [129]	Longitudinal study	13	36.6 years (median) (range: 18–59)	CMAP, MUNE	Monitoring	CMAP and SMUP increased during nusinersen treatment in ambulatory patients showing stable muscle contraction and improved motor function scores
Elsheikh et al., 2021 [128]	Longitudinal study	19	39.7 years ± 13.9 (mean ± SD) (range: 21.3–64.8)	CMAP, MUNE	Monitoring	CMAP and single motor unit potential sizes increased despite stable motor function scores in non-ambulatory patients treated with nusinersen
Schneider et al., 2021 [125]	Longitudinal study	15 patients, 15 controls	36 years (median) (interquartile range: 26–52)	MScanFit, CMAP	Monitoring	The number of motor units increased in distal muscles in ambulatory patients only during nusinersen treatment, despite unchanged motor function scores; CMAP was unchanged
Vacchiano et al., 2024 [126]	MScanFit study	23 patients, 12 controls	24 years (median) (range: 16–39)	MScanFit MUNE	Prognostic	CMAP and MUNE correlated with clinical severity; measures of collateral reinnervation (median amplitude and LSMUP) were lower in walkers compared to sitters
Querin et al., 2018 [132]	Cross-sectional study	19 patients, 16 controls	43.32 ± 14.09 years (mean ± SD)	MUNIX, MUSIX	Monitoring	MUNIX values were significantly decreased in SMA type III and IV; MUSIX was increased, suggesting active reinnervation
Otto et al., 2020 [134]	Cross-sectional MRI study	31 patients, 20 controls	29.6 years (median) (range: 7.6–73.9)	Quantitative MRI	Monitoring	Increased fat fraction and altered DTI metrics, which correlated with motor function scores and muscle strength, indicating muscle atrophy
Barp et al., [138]	Case study	2	45 and 47 years old	MRI	Monitoring	DTI showed improved muscle fiber organization during nusinersen treatment, despite stable motor scores

*CMAP* Compound muscle action potential, *DTI* Diffusion tensor imaging, *MUNE* Motor unit number estimation, *MUNIX* Motor unit number index, *MUSIX* Motor unit size index, *SD* Standard deviation, *SMA* Spinal muscular atrophy

### Case study 5

E. was a 25 year-old patient diagnosed with SMA type III at 4 years of age due to progressive difficulty walking and muscle weakness. The patient had four copies of *SMN2* and had lost ambulation at age 16, now using a wheelchair and requiring non-invasive ventilation (NIV) at night. There were no swallowing difficulties, but the patient reported limited mouth opening with fatiguability during chewing and significant trunk fatiguability requiring assistance for daily activities. Neurophysiological studies at baseline reflected the chronic denervation expected in a long-standing, wheelchair-bound SMA patient: following ulnar nerve stimulation, CMAP amplitude was severely reduced at 1.2 mV (normal > 4 mV), and MUNE showed only 8 motor units (normal > 40), indicating extensive motor neuron loss. However, evidence of chronic collateral reinnervation was present with increased A50 (3.2 mV) and LSMUP (6.8 mV), demonstrating that surviving motor neurons had enlarged their territories over time. NF-L levels were 18 pg/ml, within normal ranges for adults and therefore not contributory to clinical decision-making. Treatment was chosen after discussing delivery methods, clinical trial evidence in adults, and realistic expectations. At 14 month follow-up, neurophysiological monitoring showed modest but meaningful improvements: CMAP increased to 1.9 mV and MUNE improved to 11 motor units, suggesting some preservation or recovery of motor neuron function. Parameters reflecting reinnervation remained elevated, indicating ongoing compensatory mechanisms. Clinically, while Hammersmith Functional Motor Scale – Expanded (HFMSE) and Revised Upper Limb Module (RULM) scores remained stable (representing disease stabilization), the patient reported significant improvements in trunk fatiguability and endurance during daily activities, requiring less assistance for transfers and prolonged sitting. Respiratory function remained stable on NIV. This case demonstrates how, in chronic adult SMA, even modest neurophysiological improvements can translate into meaningful functional benefits, particularly in fatigue-related symptoms that are not captured by standard outcome measures, illustrating the complementary value of objective neurophysiological monitoring and patient-reported outcomes in this population.

## Future directions

Although we have examined the most informative biomarkers currently available for capturing the evolving complexity and phenotypic heterogeneity observed in SMA, several practical constraints limit their widespread implementation in routine clinical practice. Neurophysiological assessments demand specialized expertise and active patient cooperation, factors that may not always be readily available. Furthermore, advanced imaging modalities face significant accessibility challenges, as equipment availability during routine clinic visits remains inconsistent, while the associated costs present substantial burdens for healthcare systems and insurance providers alike.

Emerging research directions encompass several promising biomarker categories that warrant continued investigation. Direct quantification of SMN protein levels and comprehensive *SMN* transcript analysis offer the potential to monitor therapeutic targets more precisely, thereby providing enhanced insights into treatment efficacy mechanisms [149–154]. Concurrently, neuroinflammatory biomarkers – particularly cytokines associated with microglial activation and astrocytic inflammatory responses – may yield valuable perspectives regarding disease progression trajectories and therapeutic responsiveness [155, 156]. This approach appears especially relevant given the expanding recognition of neuroinflammatory processes within SMA pathophysiology.

The field of microRNA research has undergone extensive examination [53, 92, 105], although their clinical utility remains incompletely defined, and their translation into routine practice continues to face obstacles. Nevertheless, emerging evidence demonstrates altered expression patterns across various individual miRNAs and distinct miRNA classes, suggesting considerable potential for monitoring both disease progression and treatment responses. Chen and colleagues documented consistent miR34 downregulation throughout disease advancement [157], whereas investigations by multiple research groups revealed elevated serum concentrations of miR-9, miR-132, and miR-206 in SMA patients relative to healthy control populations [158, 159]. Cerebrospinal fluid analyses have further demonstrated increased levels of these miRNAs, alongside miR-218 and miR-23a, following nusinersen administration [160]. Notably, lower baseline CSF concentrations of miR-206 and miR-133a-3p correlated with superior therapeutic responses to nusinersen treatment [161]. To our knowledge, no data are available regarding the use of these biomarkers in patients treated with risdiplam.

Contemporary high-throughput technological advances have substantially broadened our comprehension of molecular alterations characteristic of SMA [162]. Comprehensive RNA sequencing investigations utilizing both cerebrospinal fluid and serum samples, complemented by single-cell RNA sequencing and spatial transcriptomics methodologies, have unveiled intricate gene expression modification patterns. These studies reveal that certain molecular markers demonstrate disease-specificity, others exhibit treatment-specific characteristics, while additional markers display treatment-dependent modifications, collectively establishing novel pathways for biomarker development [163, 164]. Proteomic investigations have similarly demonstrated SMA-specific protein modification patterns. The comprehensive work by Panicucci and colleagues successfully identified severity-dependent treatment effects, establishing distinct molecular signatures within cerebrospinal fluid across different SMA subtypes [165]. Additional proteomic research has documented altered concentrations of specific proteins in cerebrospinal fluid samples obtained from nusinersen-treated patients [166]. Remarkably, only the investigation conducted by Pant et al. has systematically examined neuroinflammatory marker alterations in both plasma and cerebrospinal fluid specimens from pediatric patients receiving AVXS-101 therapy, potentially identifying novel treatment-specific pathways [167].

Metabolomic research has contributed essential insights regarding altered biochemical pathways characteristic of SMA pathophysiology. Lu and colleagues identified metabolic markers significantly associated with disease severity within cerebrospinal fluid samples, establishing their potential utility as prognostic indicators [168]. Similarly, Saffari et al. characterized distinctive metabolic signatures present in plasma and urine specimens from SMA patients, successfully differentiating between early-onset and late-onset disease variants [169]. Limited investigations have examined metabolic consequences of nusinersen therapy within cerebrospinal fluid samples, revealing longitudinal effects on amino acid metabolic pathways [166, 170].

### Biomarker integration

Contemporary clinical practice increasingly recognizes that no single biomarker possesses sufficient capability to guide comprehensive personalized treatment strategies, acknowledging that individualized approaches require multifaceted assessment frameworks rather than uniform methodologies. Consequently, a combination of clinical observations and laboratory findings—incorporating neurophysiological assessments, neurofilament measurements, and potentially imaging studies in selected cases—appears most likely to inform evidence-based clinical decision-making. Individual biomarkers demonstrate insufficient capacity for comprehensively characterizing disease progression trajectories or accurately predicting treatment outcomes, particularly when considering the diverse age-stratified patient populations and heterogeneous SMA phenotypic presentations encountered in clinical practice.

Therefore, prioritizing the integration of multiple biomarkers within carefully designed combination panels represents a fundamental requirement for developing multidimensional assessment frameworks. Such approaches could be systematically adapted according to individual patient characteristics and specific clinical needs. The strategic combination of genetic assessments with neurophysiological measurements, supplemented by neurofilament quantification and age-appropriate imaging studies, provides substantially enhanced understanding of disease severity and potential progression patterns, thereby supporting more informed therapeutic decision-making processes.

Integrating these established biomarker approaches with novel molecular markers emerging from transcriptomic, proteomic, and metabolomic investigations within sophisticated multidimensional algorithms holds considerable promise for developing more robust, personalized, and sensitive disease-monitoring instruments. Machine learning methodologies and advanced analytical platforms prove invaluable not only for biomarker discovery and validation processes but also for facilitating the integration of multiple biomarkers to enhance patient stratification according to disease severity, progression potential, and treatment responsiveness [165, 171, 172].

## Conclusion

**Fig. 1:**
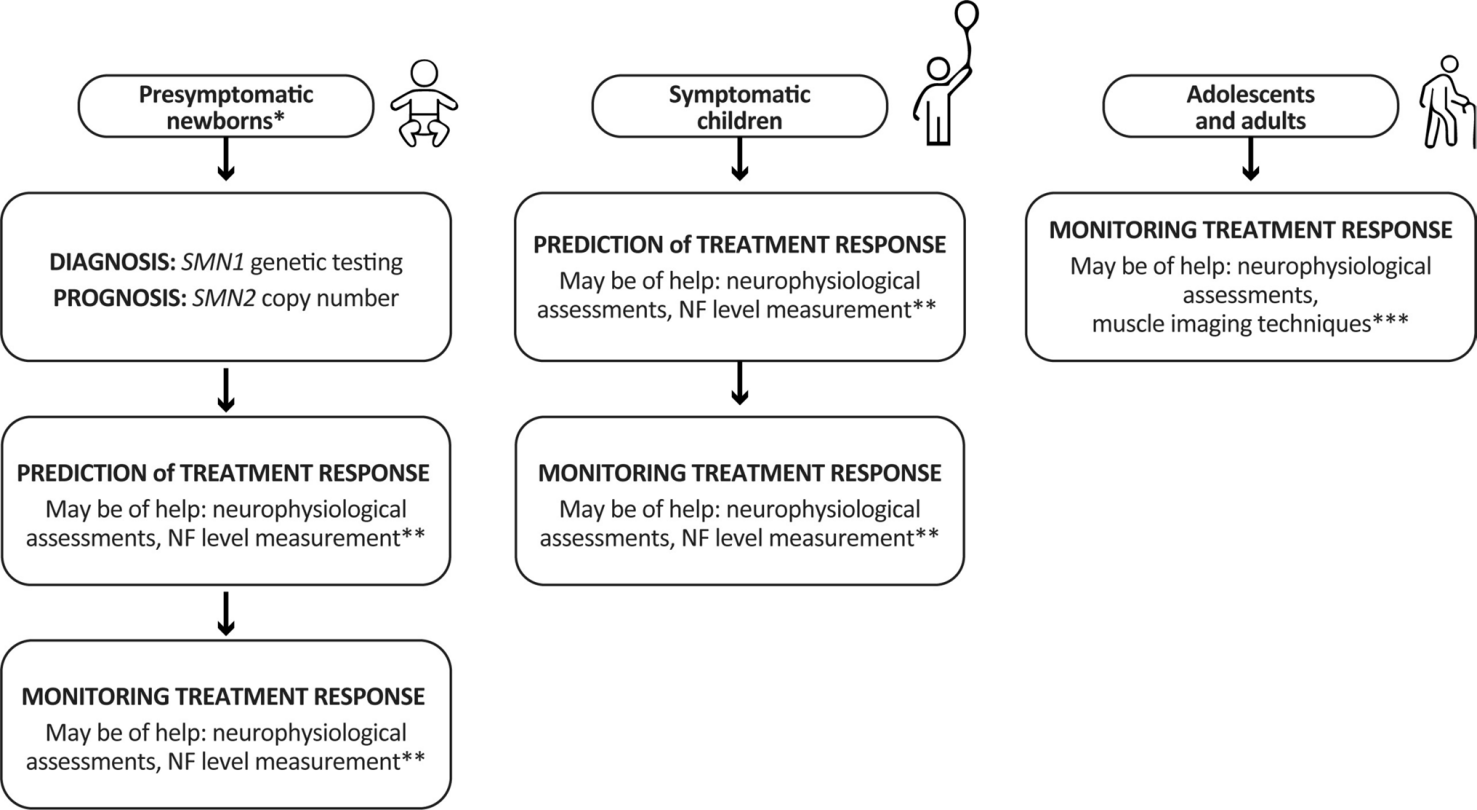
Flowchart illustrating the role of key biomarkers in SMA patients stratified by age. Newborns identified through NBS undergo genetic testing to confirm SMA via detection of *SMN1* deletion and *SMN2* copy number. The latter biomarker may help inform prognosis. In this presymptomatic stage, neurophysiological assessments and NF level measurements may be of help to predict treatment response. Following treatment initiation, these biomarkers, along with clinical outcome measures, may be used to monitor therapeutic effectiveness. In symptomatic children, in whom genetic testing is already available and treatment discussion is under way, neurophysiological markers and NF levels may serve to both predict and monitor treatment response. For adolescents and adults, neurophysiological assessments and imaging techniques may have a role for monitoring treatment efficacy over time. *Truly presymptomatic as assessed by clinical evaluation and/or Hammersmith Neonatal Neurological Examination (HNNE) and its additional module [173]. **Either in serum or CSF. ***MRI or ultrasound. *CSF* Cerebrospinal fluid, *HNNE* Hammersmith neonatal neurological examination, *MRI* Magnetic resonance imaging, *NF* Neurofilament, *SMA* Spinal muscular atrophy, *SMN* Survival motor neuron

This review illustrates how key biomarkers (*SMN2* copy number, neurophysiological measurements, NFs and imaging techniques) can offer critical insights into SMA progression and treatment outcomes at different stages of the disease (Fig. 1 and Table 4), highlighting that no single biomarker can be universally applied across all patients. A multi-biomarker approach that integrates neurophysiological, imaging, and molecular data holds promise for more sensitive, individualized monitoring and decision-making. Continuing research into novel biomarkers, along with rigorous validation and standardized methodologies, is paramount. Ultimately, these efforts will refine clinical management, enhance therapeutic interventions, and improve outcomes across the spectrum of SMA phenotypes.

**Table 4:** The role and key characteristics of selected SMA biomarkers in age-stratified patient settings

Biomarker	Age-stratified patient settings	Role	Key characteristics	Refs
*SMN1* genetic testing	All (especially newborns through NBS)	Diagnostic	Gold standard for SMA diagnosis	[55–57, 80]
*SMN2* copy number	All (especially newborns through NBS)	Prognostic	Strongest genetic predictor of disease severity; higher copy numbers generally indicate milder phenotype	[59, 60, 81]
CMAP	All	Prognostic, predictive, monitoring	Reflects motor unit integrity; can detect early degeneration and treatment response	[81, 94, 101, 103, 125, 128, 129]
MUNE	Children, adolescents/adults	Prognostic, monitoring	More sensitive than CMAP for detecting motor unit loss or remodeling	[94, 103, 126–129]
MUNIX/MUSIX	Children, adolescents/adults	Monitoring	Quantifies motor unit number and size; sensitive to chronic denervation and reinnervation	[93, 132]
NF-L/pNF-H	Newborns, children	Diagnostic, prognostic, predictive, monitoring	High levels reflect active neurodegeneration; sensitive in early disease phases	[88–90, 106, 107]
Muscle MRI	Children, adolescents/adults	Monitoring	Useful in tracking long-term muscle structural changes	[117, 134, 138]
Muscle ultrasound	Children, adolescents/adults	Monitoring	Non-invasive assessment of muscle architecture that complements MRI measurements	[114, 116, 140]

*CMAP* Compound muscle action potential, *MUNE* Motor unit number estimation, *MUNIX* Motor unit number index, *MUSIX* motor unit size index, *NF-L* Neurofilament light chain, *pNF-H* Phosphorylated neurofilament heavy chain, *SMA* Spinal muscular atrophy, *SMN* Survival motor neuron

## Data Availability

Not applicable
